# Commentary: Clinical characteristics of male prolactinoma patients mainly presenting with severe obesity and the metabolic response to dopamine agonist therapy

**DOI:** 10.3389/fendo.2024.1371468

**Published:** 2024-03-06

**Authors:** Lukas Andereggen, Emanuel Christ

**Affiliations:** ^1^ Department of Neurosurgery, Kantonsspital Aarau, Aarau, Switzerland; ^2^ Faculty of Medicine, University of Bern, Bern, Switzerland; ^3^ Department of Endocrinology, Diabetology and Metabolism, University Hospital of Basel, Basel, Switzerland

**Keywords:** prolactinoma, obesity, male, dopamine agonist, metabolic response

We read with great interest the article by Wang et al. (1) reporting the characteristics of four men with prolactinoma and severe obesity (body mass index, BMI, range: 37.9-55.9 kg/m²). Most men exhibited clinical signs of hypogonadism and presented with an adenoma ≤10mm.

After mean follow-up period of 36 ± 21 months, treatment with dopamine agonists (DAs) resulted in the control of prolactin (PRL) levels (baseline range: 72.3-273 µg/L; long-term range: 10.8-30.9). The median longitudinal change in BMI values (median ΔBMI/year) was -0.91 kg/²/year (interquartile range (IQR) of -6.11 to -0.69). Alongside, improvements in metabolic profiles were observed.

The authors’ conclude that DAs could potentially explain the weight loss alongside the metabolic advantages observed, stressing the importance of specifically screening prolactin (PRL) levels in men dealing with severe obesity.

The association between hyperprolactinemia and metabolic syndrome suggests that PRL itself acts as a regulator of body weight (2), with improvement in both BMI and metabolic profile credited to DAs therapy (3–5). In particular, individuals with prolactinoma exhibit higher BMI values and an increased incidence of obesity compared to the general population or even patients with non-functioning pituitary adenomas (6). The patients described in the cohort by Wang et al. (1) were exclusively men, aligning with the finding that males with prolactinoma exhibit higher BMIs compared to the general population (7). However, the tumor diameter in their cohort was surprisingly small. Typically, macroprolactinoma patients tend to have higher BMI values compared to patients with microprolactinomas (8). This suggests that men, experiencing more subtle nonspecific symptoms – in addition to the supposed gradual weight gain- suffer from long-term exposure to high PRL levels in contrast to women who often present with amenorrhea, an easier-to-recognize symptom prompting timely investigation and intervention (9).

The potential role of PRL in the context of obesity and metabolic syndrome becomes apparent from the lack of significant weight loss observed upon treating non-functioning adenomas, unlike it has been observed in patients with prolactinomas (6). Interestingly, a study noted significant weight loss in macroprolactinomas but not in microprolactinomas (10). Additionally, anatomical extension of large prolactinomas toward the third ventricle may cause hypothalamic compression, potentially contributing to an increased BMI rather than being solely attributed to the direct effect of hyperprolactinemia, an observation although rare in adenomas, is a well-recognized occurrence, for example, in craniopharyngiomas (11, 12). Furthermore, hypogonadism itself with subsequent reduced testosterone levels, might be linked to decreased energy levels and diminished physical activity, thus contributing to weight gain (13).

In a recent comparative study of prolactinoma patients treated either with transsphenoidal surgery (TSS) or with DAs as the primary treatment option, we observed that normalizing PRL levels led to an improvement in patients’ BMI. Notably, a significant reduction in weight was seen in the TSS cohort and a tendency for a BMI reduction in the DAs cohort (14). Considering that we noted no significant differences in the PRL levels in the long-term, it might be justified to infer that the speculated metabolic effect is likely attributable not solely to DAs themselves, but rather to the control of hyperprolactinemia and possibly the associated hypogonadism.

Considering the reported adenoma sizes in the Wang cohort, where the majority of patients have adenomas ≤10mm and are likely not infiltrating the cavernous sinus—though speculative, and noting that, at the last follow-up, all patients were still on DAs (1),—interdisciplinary discussions about upfront surgery in this specific cohort might at least be discussed, rather than committing to long-term DA therapy (9, 14–19). Whether TSS in prolactinoma patients is superior to standard care as a first-line or second-line treatment needs further investigation, particularly in an exclusive cohort of patients presenting with morbid obesity. In particular, we would like to emphasize that the primary goal is not about favoring TSS over DAs, but about controlling PRL with any means necessary to achieve a positive effect on the metabolic syndrome and BMI. Namely, in addition to managing hyperprolactinemia through surgical intervention, DA therapy, or a combination thereof, we emphasize the broader utility of DAs beyond prolactinomas (20). They have demonstrated efficacy in conditions such as Cushing’s disease and acromegaly, wherein they contribute to the reduction of cortisol and IGF-1 levels, potentially facilitating metabolic improvements (21–24). Furthermore, it is crucial to underscore the significance of considering the effects of DAs on diabetes regulation and hypertension control independently of hormonal levels (25–27). Namely, it has been shown that increased D2RS protein expression in subcutaneous adipose tissue during hyperglycemia and T2D, coupled with dopamine receptor agonists’ inhibition of adipocyte beta-adrenergic stimulation of lipolysis, may explain the observed positive effects on lipid metabolism in patients treated with bromocriptine (28). This comprehensive understanding enhances our insight into the metabolic alterations experienced by patients undergoing DA treatment.

To summarize, alongside recommending screening for PRL levels in severe obesity patients, we stress the importance of long-term correction of hyperprolactinemia and its linked hypogonadism. This might justify considering a primary surgical treatment approach as an alternative to DAs. Additionally, implementing programs for early control of weight loss interventions in this specific group of patients is crucial due to the potential high morbidity.

Acknowledging the unique and vulnerable nature of this cohort of prolactinoma patients, we commend Wang and colleagues for their insightful publication and detailed exploration of the impact of DAs on men with morbid obesity and prolactinoma. We encourage further studies to delve into the yet unexplored relationship between obesity and hyperprolactinemia, focusing on their long-term response to therapy.

## Author contributions

LA: Conceptualization, Writing – original draft, Writing – review & editing. EC: Conceptualization, Writing – review & editing.

## References

[1] Wang L Wang X Gong F Pan H Zhu H. Clinical characteristics of male prolactinoma patients mainly presenting with severe obesity and the metabolic response to dopamine agonist therapy. Front Endocrinol (Lausanne). (2023) 14:1285477. doi: 10.3389/fendo.2023.1285477 38093965 PMC10716415

[2] Shibli-Rahhal A Schlechte J. The effects of hyperprolactinemia on bone and fat. Pituitary. (2009) 12:96–104. doi: 10.1007/s11102-008-0097-3 18338266

[3] dos Santos Silva CM Barbosa FR Lima GA Warszawski L Fontes R Domingues RC. BMI and metabolic profile in patients with prolactinoma before and after treatment with dopamine agonists. Obes (Silver Spring). (2011) 19:800–5. doi: 10.1038/oby.2010.150 20559294

[4] Schwetz V Librizzi R Trummer C Theiler G Stiegler C Pieber TR. Treatment of hyperprolactinaemia reduces total cholesterol and LDL in patients with prolactinomas. Metab Brain Dis. (2017) 32:155–61. doi: 10.1007/s11011-016-9882-2 PMC5566581 27525431

[5] Byberg S Futtrup J Andreassen M Krogh J. Metabolic effects of dopamine agonists in patients with prolactinomas: a systematic review and meta-analysis. Endocr Connect. (2019) 8:1395–404. doi: 10.1530/EC-19-0286 PMC6826167 31518995

[6] Greenman Y Tordjman K Stern N. Increased body weight associated with prolactin secreting pituitary adenomas: weight loss with normalization of prolactin levels. Clin Endocrinol (Oxf). (1998) 48:547–53. doi: 10.1046/j.1365-2265.1998.00403.x 9666865

[7] Al Sabie F Tariq Z Erickson D Donegan D. Association between prolactinoma and body mass index. Endocr Pract. (2020) 27(4):312–7. doi: 10.1016/j.eprac.2020.09.001 33720014

[8] Schmid C Goede DL Hauser RS Brandle M. Increased prevalence of high Body Mass Index in patients presenting with pituitary tumours: severe obesity in patients with macroprolactinoma. Swiss Med Wkly. (2006) 136:254–8. doi: 10.4414/smw.2006.10955 16708311

[9] Andereggen L Frey J Andres RH Luedi MM Widmer HR Beck J. Persistent bone impairment despite long-term control of hyperprolactinemia and hypogonadism in men and women with prolactinomas. Sci Rep. (2021) 11:5122. doi: 10.1038/s41598-021-84606-x 33664388 PMC7933248

[10] Creemers LB Zelissen PM van ‘t Verlaat JW Koppeschaar HP. Prolactinoma and body weight: a retrospective study. Acta Endocrinol (Copenh). (1991) 125:392–6. doi: 10.1530/acta.0.1250392 1957557

[11] Choi SH Kwon BJ Na DG Kim JH Han MH Chang KH. Pituitary adenoma, craniopharyngioma, and Rathke cleft cyst involving both intrasellar and suprasellar regions: differentiation using MRI. Clin Radiol. (2007) 62:453–62. doi: 10.1016/j.crad.2006.12.001 17398271

[12] Andereggen L Hess B Andres R El-Koussy M Mariani L Raabe A. A ten-year follow-up study of treatment outcome of craniopharyngiomas. Swiss Med Wkly 148. (2018), w14521. doi: 10.4414/smw.2018.14521 29442342

[13] Mangolim AS Brito LAR Nunes-Nogueira VDS. Effectiveness of testosterone replacement in men with obesity: a systematic review and meta-analysis. Eur J Endocrinol. (2021) 186:123–35. doi: 10.1530/EJE-21-0473 34738915

[14] Andereggen L Frey J Andres RH Luedi MM Gralla J Schubert GA. Impact of primary medical or surgical therapy on prolactinoma patients’ BMI and metabolic profile over the long-term. J Clin Transl Endocrinol. (2021) 24:100258. doi: 10.1016/j.jcte.2021.100258 34195008 PMC8237353

[15] Andereggen L Christ E. Commentary: “Prolactinomas: prognostic factors of early remission after transsphenoidal surgery”. Front Endocrinol (Lausanne). (2021) 12:695498. doi: 10.3389/fendo.2021.695498 34054739 PMC8160469

[16] Andereggen L Frey J Andres RH El-Koussy M Beck J Seiler RW. Long-term follow-up of primary medical versus surgical treatment of prolactinomas in men: effects on hyperprolactinemia, hypogonadism, and bone health. World Neurosurg. (2017) 97:595–602. doi: 10.1016/j.wneu.2016.10.059 27773859

[17] Andereggen L Frey J Andres RH El-Koussy M Beck J Seiler RW. 10-year follow-up study comparing primary medical vs. Surg Ther Women prolactinomas. Endocrine. (2017) 55:223–30. doi: 10.1007/s12020-016-1115-2 27688009

[18] Andereggen L Frey J Andres RH Luedi MM El-Koussy M Widmer HR. First-line surgery in prolactinomas: lessons from a long-term follow-up study in a tertiary referral center. J Endocrinol Invest. (2021) 44(12):2621–33. doi: 10.1007/s40618-021-01569-6 PMC8572196 33847973

[19] Andereggen L Tortora A Schubert GA Musahl C Frey J Luedi MM. Prolactinomas in adolescent and elderly patients-A comparative long-term analysis. Front Surg. (2023) 10:967407. doi: 10.3389/fsurg.2023.967407 36814862 PMC9939754

[20] Biller BM Colao A Petersenn S Bonert VS Boscaro M. Prolactinomas, Cushing’s disease and acromegaly: debating the role of medical therapy for secretory pituitary adenomas. BMC Endocr Disord. (2010) 10:10. doi: 10.1186/1472-6823-10-10 20478050 PMC2887860

[21] Andersen IB Andreassen M Krogh J. The effect of dopamine agonists on metabolic variables in adults with type 2 diabetes: A systematic review with meta analysis and trial sequential analysis of randomized clinical trials. Diabetes Obes Metab. (2021) 23:58–67. doi: 10.1111/dom.14183 32869474

[22] Mehlich A Bolanowski M Mehlich D Witek P. Medical treatment of Cushing’s disease with concurrent diabetes mellitus. Front Endocrinol (Lausanne). (2023) 14:1174119. doi: 10.3389/fendo.2023.1174119 37139336 PMC10150952

[23] Chanson P. Medical treatment of acromegaly with dopamine agonists or somatostatin analogs. Neuroendocrinology. (2016) 103:50–8. doi: 10.1159/000377704 25677539

[24] Andereggen L Frey J Christ E. Long-term IGF-1 monitoring in prolactinoma patients treated with cabergoline might not be indicated. Endocrine. (2021) 72:216–22. doi: 10.1007/s12020-020-02557-1 33275185

[25] Dereje B Nardos A. Dopamine 2 agonists for the management of type 2 diabetes: a systematic review and meta-analysis. J Diabetes Metab Disord. (2023) 22:931–43. doi: 10.1007/s40200-023-01230-4 PMC10638275 37975084

[26] Murphy MB. Dopamine: a role in the pathogenesis and treatment of hypertension. J Hum Hypertens. (2000) 14 Suppl 1:S47–50. doi: 10.1038/sj.jhh.1000987 10854081

[27] Auriemma RS De Alcubierre D Pirchio R Pivonello R Colao A. Glucose abnormalities associated to prolactin secreting pituitary adenomas. Front Endocrinol (Lausanne). (2019) 10:327. doi: 10.3389/fendo.2019.00327 31191454 PMC6540784

[28] Vranic M Ahmed F Kristofi R Hetty S Mokhtari D Svensson MK. Subcutaneous adipose tissue dopamine D2 receptor is increased in prediabetes and T2D. Endocrine. (2024) 83:378–91. doi: 10.1007/s12020-023-03525-1 PMC10850013 37752366

